# Overlapping roles for PARP1 and PARP2 in the recruitment of endogenous XRCC1 and PNKP into oxidized chromatin

**DOI:** 10.1093/nar/gkw1246

**Published:** 2016-12-13

**Authors:** Hana Hanzlikova, William Gittens, Katerina Krejcikova, Zhihong Zeng, Keith W. Caldecott

**Affiliations:** Genome Damage and Stability Centre, School of Life Sciences, University of Sussex, Falmer, Brighton, BN1 9RQ, UK

## Abstract

A critical step of DNA single-strand break repair is the rapid recruitment of the scaffold protein XRCC1 that interacts with, stabilizes and stimulates multiple enzymatic components of the repair process. XRCC1 recruitment is promoted by PARP1, an enzyme that is activated following DNA damage and synthesizes ADP-ribose polymers that XRCC1 binds directly. However, cells possess two other DNA strand break-induced PARP enzymes, PARP2 and PARP3, for which the roles are unclear. To address their involvement in the recruitment of endogenous XRCC1 into oxidized chromatin we have established ‘isogenic’ human diploid cells in which *PARP1* and/or *PARP2*, or *PARP3* are deleted. Surprisingly, we show that either PARP1 or PARP2 are sufficient for near-normal XRCC1 recruitment at oxidative single-strand breaks (SSBs) as indicated by the requirement for loss of both proteins to greatly reduce or ablate XRCC1 chromatin binding following H_2_O_2_ treatment. Similar results were observed for PNKP; an XRCC1 protein partner important for repair of oxidative SSBs. Notably, concentrations of PARP inhibitor >1000-fold higher than the IC50 were required to ablate both ADP-ribosylation and XRCC1 chromatin binding following H_2_O_2_ treatment. These results demonstrate that very low levels of ADP-ribosylation, synthesized by either PARP1 or PARP2, are sufficient for XRCC1 recruitment following oxidative stress.

## INTRODUCTION

Single-strand breaks (SSBs) are one of the commonest lesions in DNA, arising at a frequency of tens-of-thousands per cell per day (1,2). One major source of SSBs are reactive oxygen species that generate DNA breaks directly by attack of deoxyribose and indirectly by triggering the excision repair of oxidized DNA bases and abasic sites. An early step in the repair of SSBs is the activation of poly (ADP-ribose) polymerases (PARPs); enzymes that covalently modify themselves and other proteins at the site of the break with mono and/or poly (ADP-ribose) and thereby serve as molecular SSB sensors (3–5). Poly (ADP-ribose) (PAR) is then bound by X-ray repair cross-complementing protein 1 (XRCC1), a molecular scaffold protein that interacts with, stabilizes and stimulates multiple enzymatic components of SSB repair and accelerates the overall process (6–9). One of the most important XRCC1 protein partners is DNA polynucleotide kinase phosphatase (PNKP) (10,11). PNKP is a dual function 5΄-DNA kinase and 3΄-DNA phosphatase that can convert oxidative DNA termini into canonical 5΄-phosphate and 3΄-hydroxyl termini that can support DNA gap filling and DNA ligation (12,13). The importance of this activity is illustrated by existence of neurological diseases in which PNKP is mutated (14–17).

The first PARP to be identified was PARP1 (ADPRT1), a 113 KDa enzyme that is responsible for ∼85–95% of the total cellular PARP activity triggered in response to DNA breaks (18). Subsequently, following the observation of residual PAR synthesis in *Parp1^-/-^* mouse embryonic fibroblasts (MEFs) treated with high doses of damaging agents, PARP2 (ADPRT2) was identified (18,19). More recently we, and others, identified PARP3 (ADPRT3) as a third ADP-ribosyl transferase (ADPRT) that is stimulated by DNA breaks (20–23). PARP1, PARP2 and PARP3 share ∼60% homology within their catalytic and tryptophan-glycine-arginine (WGR) domains, but diverge at their N-termini. The N-terminal region of PARP1 is comprised of ∼500 amino acids and includes three zinc finger domains, two of which promote binding to DNA breaks and a third that is believed to trigger stimulation of catalytic activity by up to ∼500-fold. PARP2 and PARP3 lack these zinc finger domains and instead possess shorter N-terminal regions of ∼78 and 40 amino acids, respectively, the functions of which are poorly understood. In contrast to PARP1, PARP2 and PARP3 are reliant on their WGR domains for DNA binding, perhaps explaining their lower catalytic activity.

Despite a great deal of interest in the precise roles of PARP enzymes in DNA repair their relative contribution to specific DNA repair processes remains unclear. Previous studies employing overexpressed GFP-tagged or RFP-tagged XRCC1 have demonstrated that the re-localization of these fusion proteins to focal sites of laser micro-irradiation or chromatin oxidized by hydrogen peroxide (H_2_O_2_) is largely or entirely dependent upon PARP1 (24–27). However, the overexpression of tagged XRCC1 might not accurately reflect the behaviour of endogenous XRCC1. Moreover, the role of PARP1 in promoting XRCC1 recruitment to sites of DNA damage has recently been challenged (28–31). Consequently, we have now generated *PARP1^-/-^, PARP2^-/-^, PARP1^-/-^/PARP2^-/-^* and *PARP3^-/-^* diploid human hTERT RPE-1 cell lines using CRISPR-Cas9 technology and developed high-content imaging approaches to measure the relative activity and impact of PARP1, PARP2 and PARP3 on the recruitment of endogenous XRCC1 into oxidized human chromatin. Surprisingly, we find that deletion of PARP1 alone does not dramatically impact on XRCC1 recruitment, despite the deletion of this protein reducing total ADP-ribosylation by ∼4- to 5-fold. Indeed, loss of both PARP1 and PARP2 was required to greatly reduce or ablate chromatin binding by endogenous XRCC1. Moreover, similar results were observed for endogenous PNKP, the recruitment of which was dependent on XRCC1. Consistent with these data, we show that relatively small amounts of ADP-ribosylation are required for recruitment of endogenous XRCC1 into chromatin following DNA oxidation, explaining the ability of PARP2 to substitute for PARP1.

## MATERIALS AND METHODS

### Antibodies and chemicals

The antibodies used in this study were the rabbit polyclonals anti-XRCC1 (Millipore; ABC738), anti-PARP2 (Active Motif; 39743), anti-PARP3 (4699; a kind gift from F. Dantzer), anti-PNKP (SK3195) (32), anti-poly (ADP-ribose) (Trevigen; 4336), rabbit Fc-fused anti-pan-ADP-ribose binding reagent (Millipore; MABE1016) and the mouse monoclonals anti-PARP1 (Serotec; MCA1522G), anti-poly (ADP-ribose) 10H (Enzo; ALX-804-2), anti-nucleophosmin (B23) (Invitrogen; 325200) and anti-actin (Sigma; A4700). The secondary antibodies employed for Western blotting were HRP-conjugated goat anti-rabbit (Bio-Rad; 170-6515) and goat anti-mouse (Bio-Rad; 170-6516) and for indirect immunofluorescence were goat anti-mouse or anti-rabbit Alexa 488 (Invitrogen; A11001 and A31628), goat anti-mouse Alexa 555 (Invitrogen; A21422) and donkey anti-mouse Alexa 647 (Invitrogen; A39571). 8.8 M H_2_O_2_ was obtained from Fischer Scientific. Olaparib (S1060) was purchased from Selleckchem and KU0058948 hydrochloride from Axon. Both PARP inhibitors were dissolved in dimethyl sulfoxide (Sigma) to a working concentration of 10 mM.

### Cell culture, treatment and siRNA transfection

Human hTERT RPE-1 cells obtained from ATCC were cultured in Dulbecco's Modified Eagle's Medium (DMEM/F12; Sigma) supplemented with 10% fetal calf serum (FCS) and 0.01 mg/ml hygromycin B at 37°C and 5% CO_2_. Human U2OS cells and MEFs from wild type or *Parp1^-/-^* mice (33) were cultured in DMEM (Gibco) containing 10% FCS, 2 mM glutamine and the antibiotics *penicillin* (100 units/ml) and *streptomycin* (100 μg/ml) at 37°C and 5% CO_2_. Wild-type primary human fibroblasts (1BR) and primary human fibroblasts from a *PNKP*-mutated patient (15) were cultured in Minimum Essential Media (Gibco) containing 15% FCS, 2 mM glutamine and the antibiotics penicillin (100 units/ml) and streptomycin (100 μg/ml) at 37°C and 5% CO_2_. Where indicated, cells were treated with H_2_O_2_ diluted in phosphate buffered saline (PBS) or serum free medium at the indicated concentrations immediately prior to use for 7 min at room temperature (RT) or 10 min on ice. PARP inhibitors (Olaparib or KU0058948) were employed where indicated at a final concentration of 1 μM or 10 μM and were added to the cells 1 h prior to and during H_2_O_2_ treatment. Non-targeting siRNA (ON-TARGETplus, Dharmacon), siPARP2 or siPARP3 SMARTpool (Dharmacon) were reverse-transfected into the cells using Lipofectamine^®^ RNAiMAX (Invitrogen) according to the manufacturer's instructions. All experiments were carried out 72 h post-transfection, at the observed peak of target protein depletion.

### Generation of *PARP1^-/-^, PARP2^-/-^, PARP1^-/-^/PARP2^-/-^, PARP3^-/-^* and *XRCC1^-/-^* cells

Human hTERT RPE-1 gene edited cell lines were prepared using Cas9 and guides identified using either E-CRISP (http://www.e-crisp.org/E-CRISP/) or CRISPRdirect (http://crispr.dbcls.jp). For XRCC1, the 21-mer Tru-guide (34) sequences were 5΄-GACACGACAUGGCGGAGGCGG-3΄ and 5΄-CCGCCUCCGCCAUGUCGUGUC-3΄ (PAM underlined) and spanned nucleotides 12–32 of the human *XRCC1* ORF. The 58-mer synthetic oligonucleotides XCr2F: 5΄-TTTCTTGGCTTTATATATCTTGTGGAAAGGACGAAACACCGACACGACATGGCGGAGG-3΄ and XCr2R: 5΄-GACTAGCCTTATTTTAACTTGCTATTTCTAGCTCTAAAACCCTCCGCCATGTCGTGTC-3΄ (Eurofins) encoding the 18 bp Tru-guide sequence (underlined) minus the PAM were annealed and extended into a 98-mer double-stranded fragment using Phusion polymerase (NEB) which was then subcloned into the guide RNA vector (Addgene; #41824) using Gibson Assembly (NEB).

For PARP1 deletion in RPE-1 cells we used the 22-mer Tru-guide sequences 5΄-GAAGGUGGGCCACUCCAUCCGG-3΄ and 5΄-CCGGAUGGAGUGGCCCACCUUC-3΄ (PAM underlined) spanning nucleotides 174–195 of the human *PARP1* ORF. For these experiments, the synthetic 59-mers PARP1-4F:5΄-TTTCTTGGCTTTATATATCTTGTGGAAAGGACGAAACACCGAAGGTGGGCCACTCCATC-3΄ and PARP1-4R: 5΄-GACTAGCCTTATTTTAACTTGCTATTTCTAGCTCTAAAACGATGGAGTGGCCCACCTTC-3΄ encoding the 19 bp Tru-guide (underlined) minus the PAM were subcloned as above. For PARP1 deletion in U2OS cells, we used the 20-mer Tru-guide sequences 5΄-GCACCCUGACGUUGAGGUGG-3΄ and 5΄-CCACCUCAACGUCAGGGUGC-3΄ (PAM underlined) spanning nucleotides 195–214 of the human *PARP1* ORF. For these experiments, the synthetic 57-mers PARP1-2F: 5΄-TTTCTTGGCTTTATATATCTTGTGGAAAGGACGAAACACCGCACCCTGACGTTGAGG-3΄ and PARP1-2R: 5΄-GACTAGCCTTATTTTAACTTGCTATTTCTAGCTCTAAAACCCTCAACGTCAGGGTGC-3΄ encoding the 17 bp Tru-guide (underlined) minus the PAM were subcloned as above. For PARP2, the 20-mer ‘Tru-guide΄ sequences were 5΄-GCAUCUACGAGUUUUCUUGG-3΄ and 5΄-CCAAGAAAACUCGUAGAUGC-3΄ (PAM underlined) and spanned nucleotides 107–126 of the human *PARP2* ORF. The synthetic 57-mers PARP2-F: 5΄-TTTCTTGGCTTTATATATCTTGTGGAAAGGACGAAACACCGCATCTACGAGTTTTCT-3΄ and PARP2-R: 5΄-GACTAGCCTTATTTTAACTTGCTATTTCTAGCTCTAAAACAGAAAACTCGTAGATGC-3΄ encoding the 17 bp Tru-guide sequence (underlined) minus the PAM were subcloned as above. For PARP3, the 20-mer ‘Tru-guide’ sequences were 5΄-GAUUAUGCGCUUCUCUGCGG-3΄ and 5΄-CCGCAGAGAAGCGCAUAAUC-3΄ (PAM underlined) and spanned nucleotides 119–138 of the human PARP3 ORF. The synthetic 57-mers PARP3-1F: 5΄-TTTCTTGGCTTTATATATCTTGTGGAAAGGACGAAACACCGATTATGCGCTTCTCTG-3΄ and PARP3-1R: 5΄-GACTAGCCTTATTTTAACTTGCTATTTCTAGCTCTAAAACCAGAGAAGCGCATAATC-3΄ encoding the 17 bp Tru-guide sequence (underlined) minus the PAM were subcloned as above.

Generation of *PARP1^-/-^*/PARP2^-/-^ RPE-1 cells was carried out by targeting *PARP2* in *PARP1^-/-^* RPE-1 cells. The 20-mer ‘Tru-guide’ sequences targeting PARP2 in this case were 5΄-GAGGAUUGUAUUCGGGCUGG-3΄ and 5΄-CCAGCCCGAAUACAAUCCUC-3΄ (PAM underlined) and spanned nucleotides 11821–11841 of the human *PARP2* ORF. The synthetic 57-mers PARP2-B-F: 5΄-TTTCTTGGCTTTATATATCTTGTGGAAAGGACGAAACACCGAGGATTGTATTCGGGC-3΄ and PARP2-B-R: 5΄-GACTAGCCTTATTTTAACTTGCTATTTCTAGCTCTAAAACGCCCGAATACAATCCTC-3΄ encoding the 17 bp Tru-guide (underlined) minus the PAM were subcloned as above.

For gene editing, hTERT RPE-1 or U2OS cells were co-transfected with the appropriate guide RNA construct indicated above and a Cas9 expression construct (Addgene; #41815) using a NEON Transfection System (Invitrogen). Twenty four hours later, the transfected cells were selected in medium containing 0.5 mg/ml G418 for 5 days and then subcloned into 96-wells plates. Once at sufficient cell density the subclones were analysed for presence of the target protein by indirect immunofluorescence (PARP1, PARP2, XRCC1) or by Western blotting (PARP3). The absence of the targeted protein in cell clones selected as above was confirmed by Western blotting and one clone of each genotype was chosen for further analysis (clone #G7 for *PARP1^-/-^*, #A1 for *PARP2^-/-^*, #E6 for *PARP1^-/-^/PARP2^-/-^*, #20 for *PARP3^-/-^* and #3 for *XRCC1^-/-^* cells).

### Confirmation of gene editing by sanger sequencing

Genomic DNA was purified from WT, *PARP1^-/-^, PARP2^-/-^, PARP3^-/-^, PARP1^-/-^/PARP2^-/-^* and *XRCC1^-/-^* RPE-1 cells using Extract-N-Amp Tissue PCR kit (Sigma) as per the manufacturer's instructions. PCR was used to amplify regions of interest surrounding the specific gRNA target loci (primer sequences can be found in Supplementary Table S1). Amplicons were cloned and expanded in pCR^®^2.1-TOPO^®^ (Invitrogen) prior to Sanger sequencing. WT RPE-1 cells were sequenced by whole exome sequencing (Source BioScience). Consensus sequences for each pCR^®^2.1-TOPO^®^ clone were aligned with the consensus sequences for the corresponding loci from WT RPE-1 exome sequencing, allowing identification of indels close to the gRNA target loci.

### SDS-PAGE and Western blotting

Collected cells were lysed in standard 1x Laemmli loading buffer, denaturated for 10 min at 95°C and sonicated for 30 s using Bioruptor^®^ Pico (Diagenode). Protein concentrations were determined using the BCA assay (Pierce). Samples were subjected to SDS-PAGE (8% or gradient gel), proteins transferred onto nitrocellulose membrane and detected by relevant specific antibodies combined with horseradish peroxidase-conjugated secondary antibodies. Peroxidase activity was detected by ECL reagent (GE Healthcare) and Amersham Hyperfilm ECL (GE Healthcare) or ImageQuant LAS-4000 system connected with high-sensitivity Super CCD camera (GE Healthcare).

### Immunofluorescence and microscopy

Cells grown on coverslips were washed with PBS and fixed with 4% paraformaldehyde in PBS 10 min at RT. After fixation, cells were washed twice with PBS, treated with ice-cold methanol/acetone solution (1:1) for 5 min, washed twice with PBS and blocked at least 30 min in 10% FCS in PBS. Incubation with the primary antibody (60 min, RT) was followed by wash (3 × 5 min in PBS) and incubation with appropriate fluorescently-labelled secondary antibody (60 min, RT). Coverslips were washed (3 × 5 min in PBS), stained with DAPI (1 μg/ml in water, 2 min) and mounted using VECTASHIELD (Vector Laboratories). To measure chromatin retention of proteins, cells were pre-extracted in cold 0.2% Triton X-100 for 2 min on ice prior to fixation as above. High-resolution microscopy of fixed samples was carried out on a Zeiss AxioObserver.Z1 microscope, equipped with oil immersion objectives (Plan-Apochromat 63x/1.4), Hamamatsu ORCA-Flash4.0 LT camera and ZEN 2 core imaging software. Automated wide-field microscopy was performed on an Olympus ScanR system (motorized IX83 microscope) with ScanR Image Acquisition and Analysis Software, 20x/0.45 (LUCPLFLN 20x PH) and 40x/0.6 (LUCPLFLN 40x PH) dry objectives and Hamamatsu ORCA-R2 digital CCD camera C10600. Total nuclear ADP-ribose fluorescence signal was quantified in the region colocalizing with DAPI. Non-nucleolar anti-XRCC1 fluorescence signal was quantified in the region colocalizing with DAPI but excluding the nucleolar region defined by B23 colabelling. Fluorescence was plotted relative to that in untreated WT cells. Where indicated, non-specific anti-XRCC1 background fluorescence was measured using *XRCC1^-/-^*cells and is indicated by a black dotted line in the relevant graphs.

### Alkaline comet assays

A total of 3 × 10^6^ cells were trypsinized, washed and resuspended in ice cold PBS. A total of 5 × 10^5^ cells were removed and stored on ice (‘undamaged’ sample). The remaining cells were then treated with H_2_O_2_ (50 μM) in PBS for 10 min on ice before mixing with complete DMEM/F12 medium prior to recovery of the cells by centrifugation and resuspension in ice cold complete DMEM/F12. A total of 5 × 10^5^ cells were removed and stored on ice (‘no repair’ sample) and the remaining cells resuspended in complete DMEM/F12 (37°C) and incubated at 37°C for the indicated repair period. At 7.5, 15 and 30 min, 5 × 10^5^ cells were removed and stored on ice. Finally, all samples were resuspended in 200 μl ice cold PBS, rapidly mixed with 200 μl 1.2% low melting point agarose in PBS and plated on 0.6% agarose-coated, frosted glass slides on ice. The agarose was allowed to solidify prior to incubation in lysis buffer (2.5 M NaCl, 100 mM EDTA, 10 mM Tris-HCl pH 10) for 1 h at 4°C. Slides were washed 3 times with 4°C H_2_O and incubated for 45 min in electrophoresis buffer (1 mM EDTA, 50 mM NaOH). Electrophoresis was carried out at 12 V for 25 min, prior to overnight neutralization with Tris-HCl (1 M). Finally, slides were stained with Tris-HCl (1 M) containing SYBR-G (1:10000) and Antifade (40 μg/ml), and imaged (Nikon Eclipse 50i). Average tail moments from 100 cells per sample were obtained using Comet Assay IV software (Perceptive Instruments).

## RESULTS

**Figure 1.**
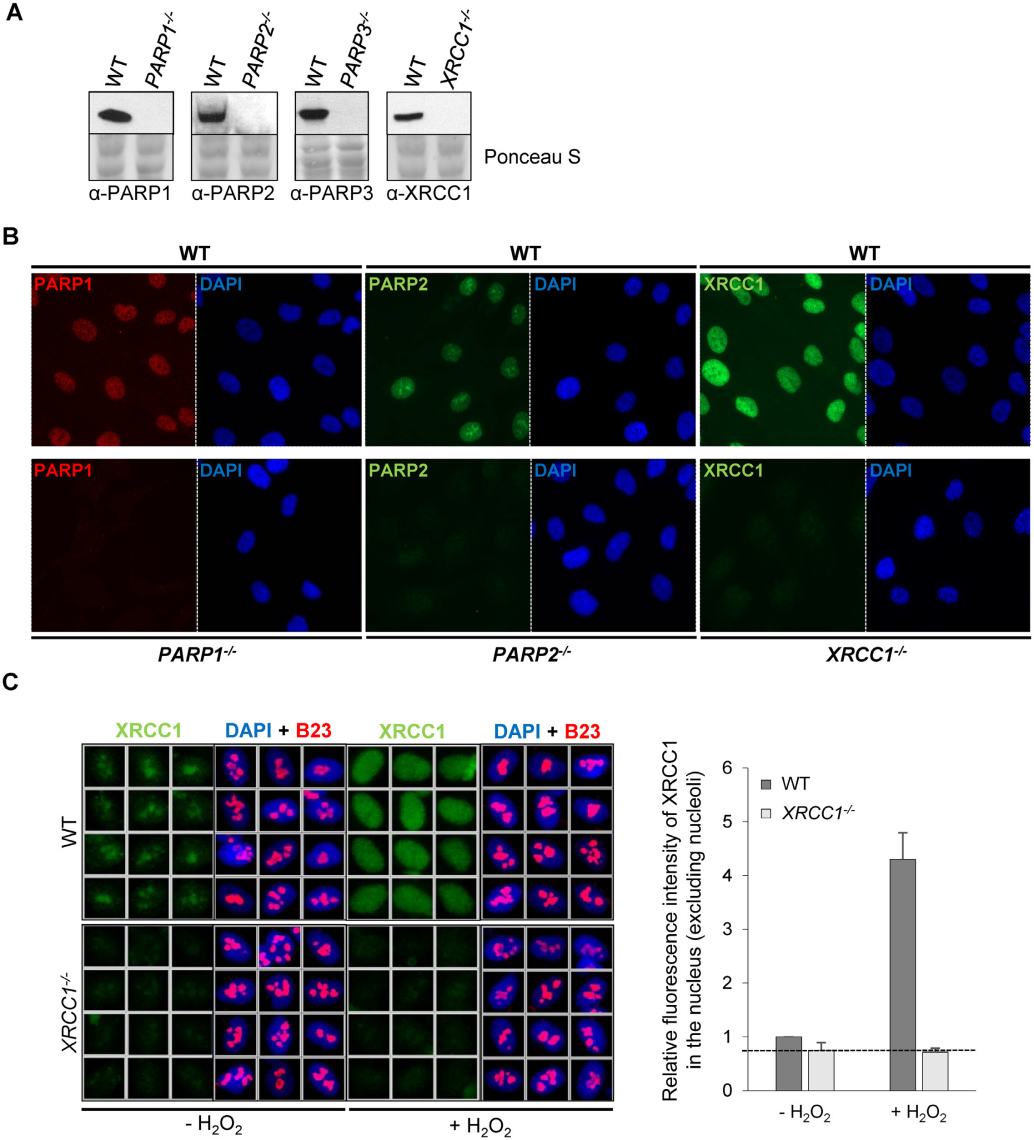
Development of *PARP1^-/-^, PARP2^-/-^, PARP3^-/-^* and *XRCC1^-/-^* RPE-1 cells and XRCC1 high-content imaging. Wild type (WT), *PARP1^-/-^, PARP2^-/-^, PARP3^-/-^* and *XRCC1^-/-^* RPE-1 clonal cell lines were analysed for loss of the targeted protein by (**A**) Western blotting and (**B**) immunofluorescence. Note that the PARP3 antibody available to us was not suitable for immunofluorescence. (**C**) *Left*, representative ScanR images of WT and *XRCC1^-/-^* RPE-1 cells non-treated or treated with 1 mM hydrogen peroxide (H_2_O_2)_ for 10 min and pre-extracted with detergent prior to fixation and immunostaining for XRCC1 (green), the nucleolar marker B23 (red) and counterstaining with DAPI (blue). *Right*, quantification of detergent-insoluble anti-XRCC1 signal (excluding nucleolar XRCC1 signal) from >1000 cells per sample using Olympus ScanR analysis software. Data are the mean (±SEM) of three independent experiments. The black dotted line denotes non-specific anti-XRCC1 background signal, defined as the residual signal in *XRCC1^-/-^* RPE-1 cells.

To examine the respective roles of PARP1, PARP2 and PARP3 on the recruitment of endogenous XRCC1 at sites of DNA damage we generated a set of diploid human hTERT RPE-1 cell lines (denoted RPE-1 for simplicity) in which these four proteins were deleted individually using CRISPR/Cas9-mediated genome editing. Individual clones in which the relevant protein was absent were identified using Western blotting and immunofluorescence and a single clone of each genotype was chosen for further experiments (Figure 1A and B). That the relevant CRISPR/Cas9 target site was mutated was confirmed by PCR and Sanger sequencing (Supplementary Figure S1A).

The generation of *XRCC1^-/-^* RPE-1 cells and the identification of a specific anti-XRCC1 antibody for immunofluorescence enabled us to develop a high-content imaging approach for measuring the recruitment of endogenous XRCC1 into oxidized human chromatin. Interestingly, when soluble proteins were extracted with detergent prior to fixation and immunostaining XRCC1 was localized primarily in the nucleoli in undamaged wild-type RPE-1 cells (Figure 1C, *left*). This was not the case in *XRCC1^-/-^* RPE-1 cells, confirming that the anti-XRCC1 nucleolar signal was specific. More importantly, XRCC1 was rapidly recruited into chromatin globally across the nucleus following treatment with H_2_O_2_. H_2_O_2_ is a physiologically relevant source of oxidative stress and SSBs but induces DSBs only very poorly, with a SSB/DSB ratio of >2000/1 (35). To quantify XRCC1 recruitment into global nuclear chromatin following H_2_O_2_ treatment we employed an Olympus ScanR automated wide-field microscope with image acquisition and analysis software, and excluded signal co-localising with the nucleolar marker, nucleophosmin (B23). These data revealed that the amount of chromatin bound XRCC1 increased 5-fold in wild-type RPE-1 cells following treatment with H_2_O_2_, but did not increase above background signal in *XRCC1^-/-^* RPE-1 cells (Figure 1C, *right*).

**Figure 2.**
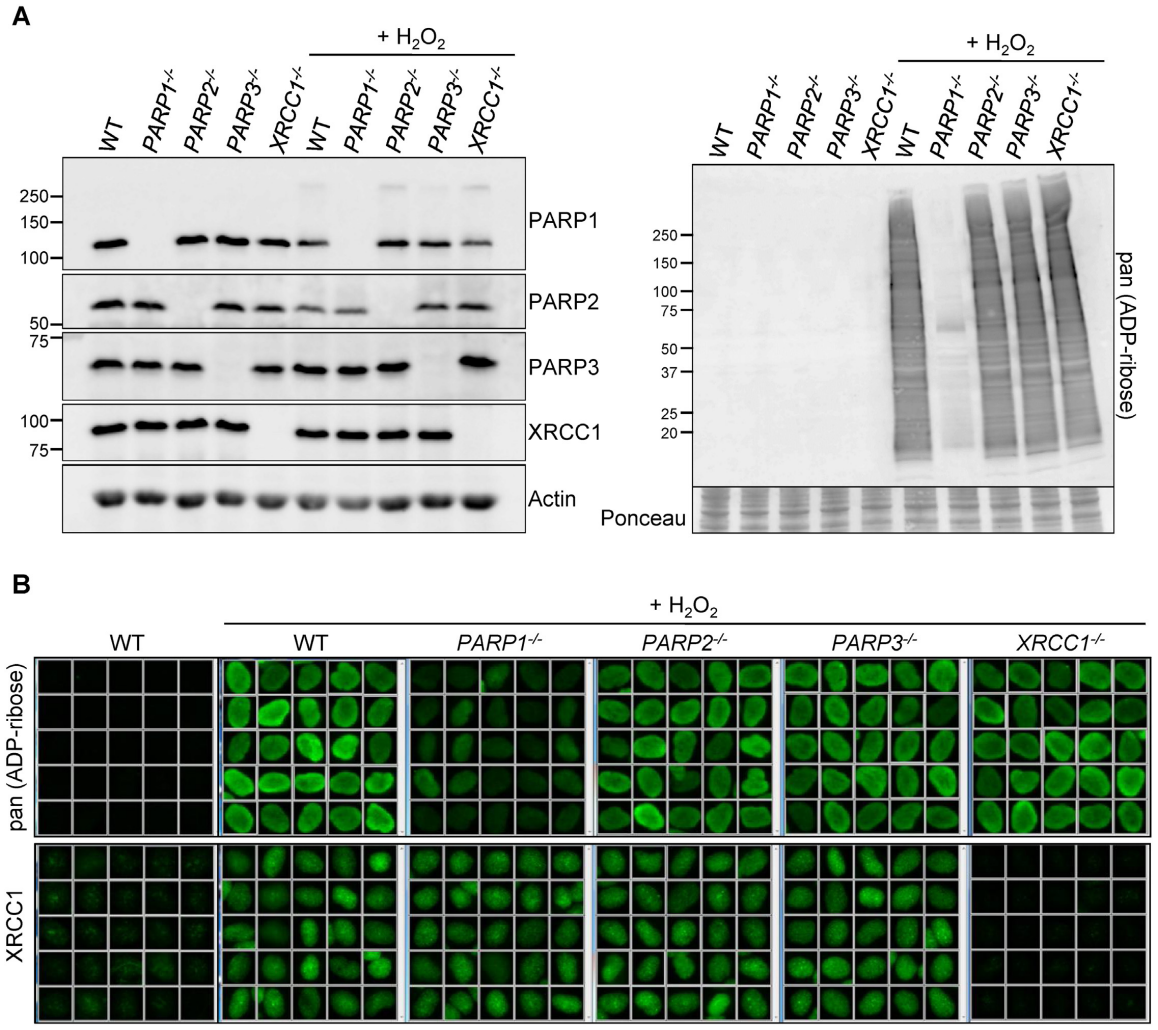
Levels of H_2_O_2_-induced ADP-ribosylation and XRCC1 recruitment into chromatin in PARP-deleted RPE-1 cells. (**A**) Levels of the indicated proteins (*left*) and ADP-ribosylated proteins (*right*) were compared in cell lysates from the indicated WT or mutant RPE-1 cells harvested before and after treatment with 400 µM H_2_O_2_ for 7 min by Western blotting using appropriate antibodies and anti-pan-ADP-ribose binding reagent. (**B**) Levels of ADP-ribosylation and chromatin-bound XRCC1 were analysed by indirect immunofluorescence in cells treated or not with H_2_O_2_ (as above) by fixation and staining with anti-pan-ADP-ribose binding reagent (*top panels*) or by detergent pre-extraction prior to fixation and staining with anti-XRCC1 antibody (*bottom panels*). Representative ScanR images are shown.

Next, we examined levels of ADP-ribosylation and XRCC1 chromatin loading in RPE-1 cells deleted of the individual PARP proteins. Importantly, only the level of the targeted PARP was affected in each of the gene-edited cell lines (Figure 2A, *left*). Only in *PARP1^-/-^* cells were the levels of ADP-ribosylation visibly reduced, as measured by Western blotting and indirect immunofluorescence (Figure 2A, *right* and B). This is consistent with previous observations demonstrating that PARP1 accounts for 80–90% of total ADP-ribosylation following DNA damage (18). In our hands, PARP1 deletion resulted in ∼75–80% reduction in total ADP-ribosylation under the conditions employed (Figures 2B and 4B). Surprisingly, however, the level of XRCC1 recruitment into oxidized chromatin was not significantly reduced by PARP1 deletion (Figures 2B and 4B). This was not an artefact of clonal selection because XRCC1 recruitment into chromatin was not measurably reduced in two other independent *PARP1^-/-^* clones (Supplementary Figure S1B and C).

**Figure 3.**
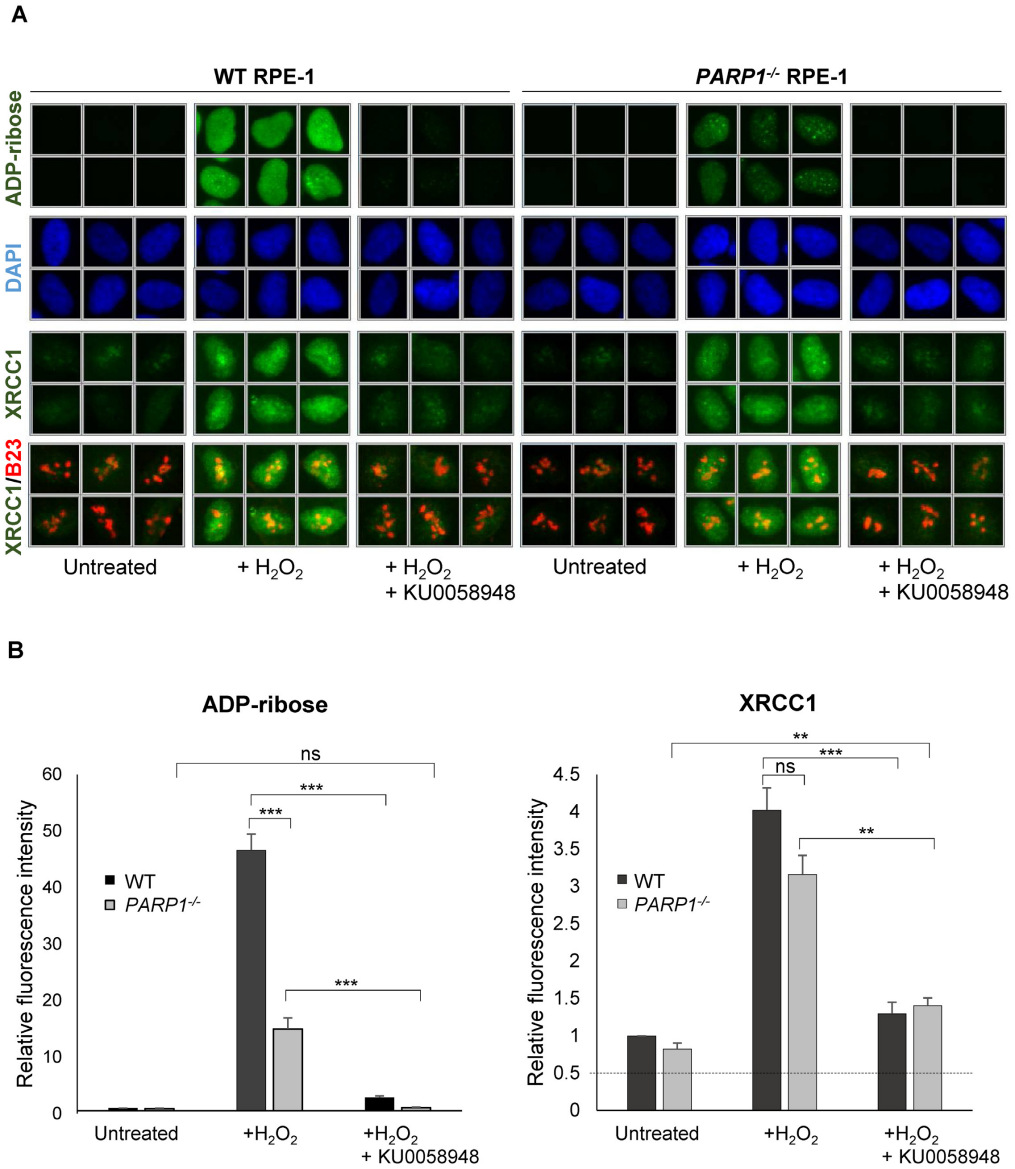
Residual recruitment of endogenous XRCC1 into oxidized chromatin in *PARP1^-/-^* RPE-1 cells is greatly reduced by PARP inhibitor. (**A**) WT and *PARP1^-/-^* RPE-1 cells were pre-incubated or not with 10 μM KU0058948 inhibitor for 1 h prior to a 7 min incubation with or without 400 μM H_2_O_2_. Cells were pre-extracted with detergent to remove non-chromatin bound proteins prior to fixation and immunostaining with the indicated antibodies or anti-pan-ADP-ribose binding reagent. Representative ScanR images are shown. (**B**) Quantification of total nuclear pan-ADP-ribose and chromatin-bound nuclear XRCC1 (excluding nucleolar XRCC1 signal) in cells treated as in panel A. Nucleoli were located using anti-B23 antibodies. All data are the mean (±SEM) of three independent experiments with >1000 cells scored per sample in each experiment. Statistical significance was assessed by two-tailed t-tests. Asterisks ** and *** indicate *P*-values of <0.01 and <0.001, respectively; ns – not significant. The black dotted line denotes non-specific anti-XRCC1 background signal, defined as the residual signal in *XRCC1^-/-^* cells stained in parallel.

The high level of XRCC1 recruitment into oxidized chromatin observed in *PARP1^-/-^* RPE-1 cells suggests that the residual ADP-ribosylation observed in these cells is sufficient for XRCC1 recruitment. Consistent with this, treatment of wild type or *PARP1^-/-^* RPE-1 cells with the PARP inhibitor KU0058948 at 10 μM ablated ADP-ribosylation and reduced H_2_O_2_-induced XRCC1 recruitment by more than 85% (Figure 3). At lower concentrations of KU0058948 (1 μM) we observed a small amount of residual ADP-ribosylation by immunofluorescence analysis, and this correlated with increased residual XRCC1 loading in chromatin (Supplementary Figure S2). It is noteworthy that the residual ADP-ribosylation remaining in cells pre-incubated with lower concentrations of KU0058948 was detected only with highly sensitive ADP-ribose detection reagents and only by immunofluorescence analysis, highlighting the importance of careful analysis when correlating the impact of PARP inhibitors on ADP-ribosylation with biological end points (Supplementary Figure S3).

Collectively, the results described above suggest that the recruitment of endogenous XRCC1 into oxidized chromatin in human diploid RPE-1 cells is largely dependent on ADP-ribosylation but does not require the presence of PARP1. To examine if this was also true in other cell types we employed wild type and *PARP1^-/-^* U2OS cells and MEFs. Indeed, XRCC1 recruitment into oxidized chromatin was not noticeably affected by PARP1 deletion in U2OS and MEFs, respectively (Supplementary Figure S4). Once again, the ability to support XRCC1 chromatin loading reflected residual levels of ADP-ribosylation in *PARP1^-/-^* cells, because both these residual levels and XRCC1 loading were ablated by incubation with PARP inhibitor (Supplementary Figure S4).

**Figure 4.**
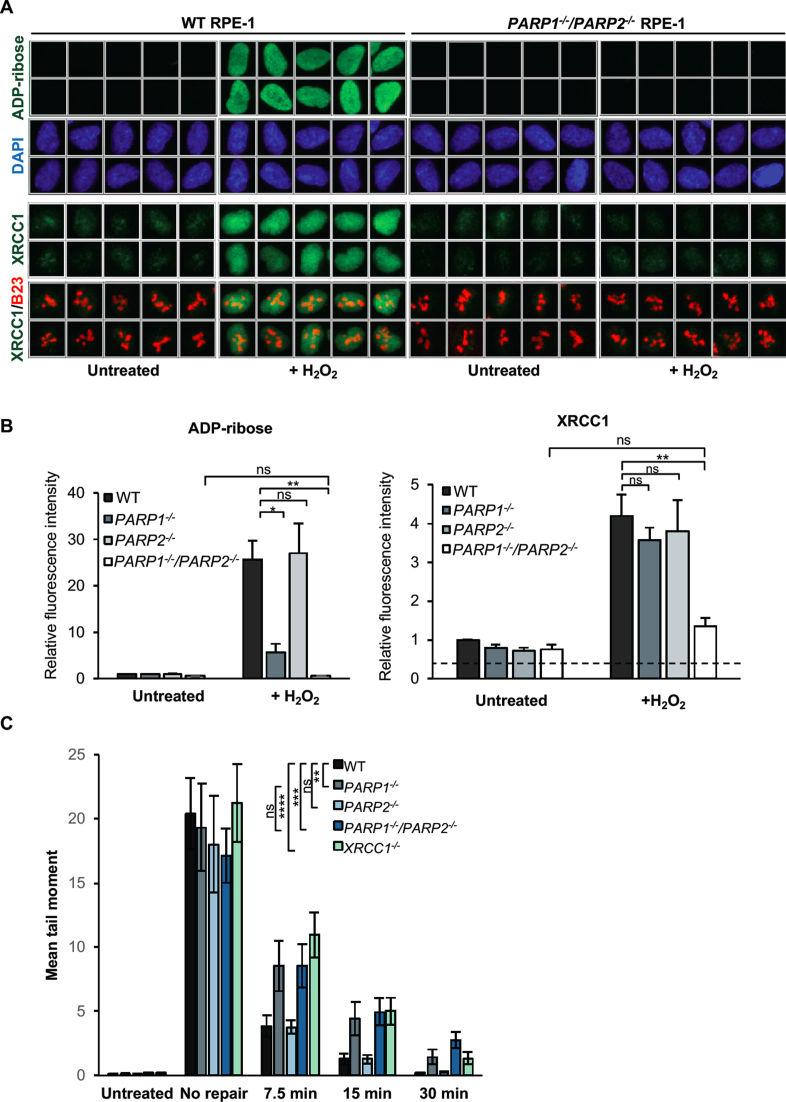
Overlapping roles for PARP1 and PARP2 in recruiting endogenous XRCC1 into oxidized chromatin. (**A**) Levels of ADP-ribosylation and chromatin-bound XRCC1 were measured by indirect immunofluorescence in WT and *PARP1^-/-^/PARP2^-/-^*cells treated or not with 400 μM H_2_O_2_ for 7 min by fixation and staining with anti-pan-ADP-ribose binding reagent and DAPI (*top panels*) or by detergent pre-extraction prior to fixation and staining with anti-XRCC1 and anti-B23 antibodies (*bottom panels*). Representative ScanR images are shown. (**B**) Quantification of total nuclear pan-ADP-ribose and chromatin bound XRCC1 (excluding nucleolar signal). The black dotted line denotes non-specific anti-XRCC1 background signal, measured by XRCC1 immunostaining in *XRCC1^-/-^* cells in parallel. All data are the mean (±SEM) of three independent experiments with >1000 cells scored per sample in each experiment. Statistical significance was assessed by two tailed t-tests. Asterisks * and ** indicate *P*-values of <0.05 and <0.01, respectively; ns – not significant. (**C**) DNA strand breakage was quantified by alkaline comet assays in indicated RPE-1 cells before, immediately after treatment with 50 μM H_2_O_2_ on ice and after the depicted repair periods in drug-free medium. Data are the average comet tail moment (an arbitrary unit-measure of DNA strand breaks) of 100 cells per sample and are the mean (±SEM) of three independent experiments. Statistically significant differences (two-way ANOVA) are indicated (**P < 0.01; ***P < 0.001; ****P < 0.0001; ns – not significant).

Given the dependence on ADP-ribosylation for recruitment of endogenous XRCC1 into oxidized chromatin, and the individual dispensability of PARP1, PARP2 and PARP3 for this process, we considered the possibility that two or more of these enzymes exhibit redundant or overlapping roles. The possible redundancy between PARP1 and PARP2 was of particular interest, because PARP2 has been reported to account for the residual poly ADP-ribosylation detected in *Parp1^-/-^* MEFs (18). Indeed, deletion of *PARP2* in *PARP1^-/-^* RPE-1 cells (denoted *PARP1^-/-^/PARP2^-/-^* cells) reduced the residual ADP-ribosylation below the level of detection under the conditions employed and reduced XRCC1 recruitment into oxidized chromatin to levels that were not significantly above background (Figure 4A and B). XRCC1 recruitment into chromatin was similarly selectively reduced in *PARP1^-/-^* RPE-1 cells by PARP2 siRNA (Supplementary Figure S5), thereby demonstrating functional overlap between PARP1 and PARP2 using two independent approaches. In contrast, loss of PARP3 alone or together with PARP1 failed to impact on XRCC1 recruitment in RPE-1 cells (Figure 2B and Supplementary Figure S5). Interestingly, PARP2 was not able to functionally replace PARP1 with respect the rate of SSBR, because the rate at which DNA breaks declined following H_2_O_2_ treatment was equally slow in *PARP1^-/-^* and *PARP1^-/-^/PARP2^-/-^* cells (Figure 4C). This is consistent with our previous findings (27), and suggests that PARP1 fulfils a second role during SSBR that is functionally distinct from PARP2.

**Figure 5.**
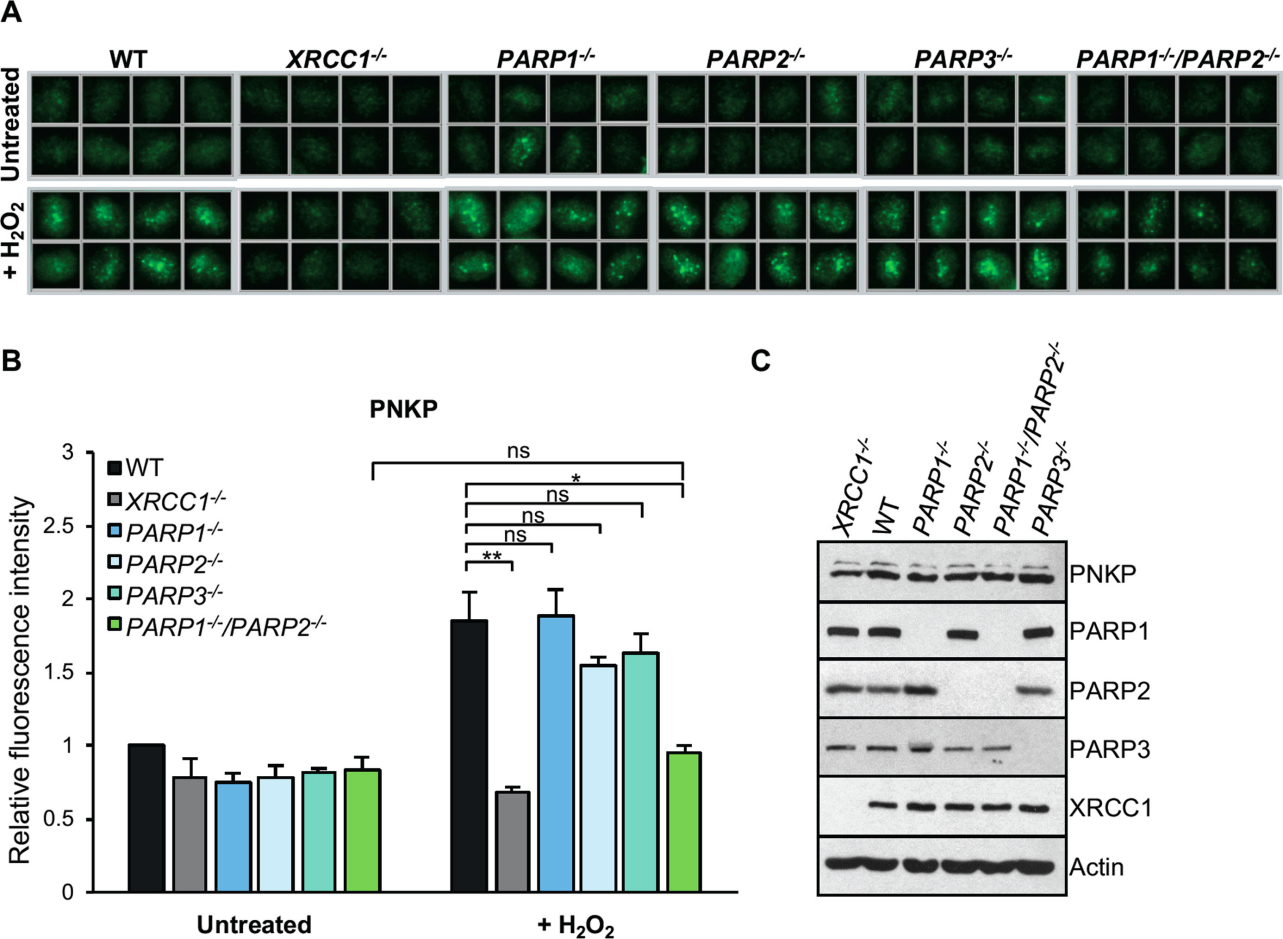
Overlapping roles for PARP1 and PARP2 in recruiting endogenous PNKP into oxidised chromatin. (**A**) Levels of chromatin-bound PNKP were analysed by indirect immunofluorescence in indicated RPE-1 cell lines untreated or treated with 400 μM H_2_O_2_ for 7 min by detergent pre-extraction prior to fixation and staining with anti-PNKP antibody. Representative ScanR images are shown. (**B**) Quantification of chromatin-bound PNKP in cells measured as above. All data are the mean (±SEM) of three independent experiments with >2000 cells scored per sample in each experiment. Statistical significance was assessed by two-tailed t-tests. Asterisks * and ** indicate *P*-values of <0.05 and <0.01, respectively; ns – not significant. (**C**) Levels of PNKP and the relevant proteins in cell extracts from RPE-1 cells of the indicated genotype.

Finally, we examined whether the functional overlap between PARP1 and PARP2 in XRCC1 recruitment extended to another component of the XRCC1-dependent SSBR pathway. For this we chose PNKP, an important DNA strand break repair enzyme reported previously to be recruited to SSBs by interaction with XRCC1 (11). Indeed, consistent with this, the level of chromatin-bound nuclear anti-PNKP staining increased 2-fold in wild type RPE-1 cells following H_2_O_2_ treatment, but failed to do so in *XRCC1^-/-^* RPE-1 cells (Figure 5A and B). That the PNKP immunostaining in these experiments was specific was confirmed using primary fibroblasts from a patient (15) in which PNKP is mutated and greatly reduced (Supplementary Figure S6A). More importantly, whereas deletion of neither *PARP1* nor *PARP2* alone significantly reduced PNKP recruitment into oxidised chromatin, co-deletion of both of these genes did so (Figure 5A and B). This did not reflect a difference in total PNKP levels because this was similar in wild type and *PARP1^-/-^/PARP2^-/-^* RPE-1 cells (Figure 5C and Supplementary Figure S6B).

In summary, we show here that the SSB sensor proteins PARP1 and PARP2 fulfil overlapping roles in promoting the recruitment of endogenous XRCC1 and PNKP into oxidized human chromatin during chromosomal SSBR, such that either enzyme can support this function.

## DISCUSSION

XRCC1 is a scaffold protein that interacts with multiple enzymatic components of SSB repair and thereby accelerates the overall process (2,8). Here, we have applied CRISPR-Cas9 technology and quantitative high-content imaging to investigate, for the first time, the role of PARP1, PARP2 and PARP3 in the recruitment of endogenous XRCC1 to sites of chromosome damage. Detergent pre-extraction suggested that, prior to exogenous DNA damage, chromatin-bound XRCC1 is located predominantly in nucleoli. This is consistent with several previous observations (24,36–38) and suggests that nucleoli are a storage site for this protein and/or that XRCC1 has an as yet undefined role in maintaining ribosomal DNA metabolism.

Importantly, we detected a 5-fold increase in the amount of detergent-insoluble XRCC1 present outside of the nucleolus following H_2_O_2_ treatment, suggesting that XRCC1 becomes distributed throughout the nuclear chromatin in response to oxidative stress. This is consistent with the observation that, under the conditions employed here, H_2_O_2_ induces 10 000–20 000 SSBs per cell (unpublished observations). Our experiments employing PARP inhibitors demonstrate unequivocally that the global recruitment of endogenous XRCC1 into oxidized chromatin required poly (ADP-ribose) polymerase activity, a notion that has been questioned recently (28–31). A surprising finding of this work, however, is that relatively little ADP-ribosylation is required for XRCC1 recruitment, compared to the total cellular level of ADP-ribosylation following H_2_O_2_ treatment. For example, to completely block ADP-ribosylation and ablate XRCC1 recruitment required a concentration of the PARP inhibitor KU0058948 (10 μM) that was 3000-fold higher than the IC50 (3.4 nM) (39). Indeed, residual levels of XRCC1 recruitment were observed at levels of ADP-ribosylation that were too low to be detected by Western blotting. Only by employing highly sensitive ADP-ribose antibodies and detection reagents in immunofluorescence experiments were we able to detect this level of ADP-ribosylation.

XRCC1 binds directly to poly (ADP-ribose) via its central BRCT1 domain, thereby enabling this scaffold protein to accumulate at sites of PARP activity (6,7). The recruitment of XRCC1 at sites of chromosomal SSBs has been reported by us and others to be dependent on PARP1, which is the most abundant and active poly (ADP-ribose) polymerase and is the primary source of poly (ADP-ribose) following oxidative stress (24–27,40). However, we have now found that deletion of PARP1 alone is unable to prevent the recruitment of endogenous XRCC1 into oxidized chromatin globally across the nucleus, suggesting that the level of ADP-ribosylation remaining in *PARP1^-/-^* cells is sufficient for XRCC1 loading into oxidised chromatin. Indeed, loss of both PARP1 and PARP2 was required to ablate H_2_O_2_-induced ADP-ribosylation and prevent loading of endogenous XRCC1 into oxidized chromatin. We suggest that this discrepancy reflects the use in previous studies of overexpressed XRCC1 and/or the measurement of XRCC1 accumulation at a limited number of focal or highly damaged sites. Whereas only PARP1 has sufficient activity to load high levels of overexpressed XRCC1 at focal sites of damage, either PARP1 or PARP2 is able to achieve this for endogenous XRCC1 at more physiological levels of oxidized chromatin induced stochastically across the genome.

This functional overlap between PARP1 and PARP2 in protein recruitment into oxidized chromatin was not restricted to XRCC1. Indeed, we observed similar results for recruitment of PNKP, an important partner protein of XRCC1 that is critical for rapid repair of oxidative DNA breaks (10,11). PNKP is a dual function 5΄-DNA kinase and 3΄-DNA phosphatase which if mutated results in human neurological disease characterized by progressive cerebellar ataxia and early onset seizures with developmental delay (14–16). It has been reported previously that XRCC1 recruits recombinant PNKP at sites of oxidative damage, but our data are the first demonstration that this is true for the endogenous protein. This is important because several recent publications have instead concluded that PNKP is recruited to DNA damage sites by alternative mechanism/s including direct binding of PNKP to DNA and/or poly (ADP-ribose) (6,29,30). However, our experiments show that most if not all chromatin binding by endogenous PNKP is XRCC1-dependent following H_2_O_2_ treatment.

Our finding that either PARP1 or PARP2 can support loading of endogenous XRCC1 and PNKP into oxidized chromatin is consistent with a functional overlap between these PARP enzymes (41). It has been shown previously that whereas both *Parp1^-/-^*and *Parp2^-/-^* mice are viable, mice lacking both enzymes die early during embryogenesis, further demonstrating a functional interplay between these enzymes (42). One prediction arising from our data is that the loss of both PARP1 and PARP2 should be necessary to reduce the rate of chromosomal SSBR. However, in our previous work we found that only PARP1 depletion reduced this rate, and that PARP2 depletion did not measurably slow SSBR even in cells in which PARP1 was depleted (27). Indeed, we observed the same result in the current work, in which we compared SSBR rates in RPE-1 cells deleted of PARP1 or PARP2 separately and together. This epistatic relationship suggests that whilst loss of both PARP1 and PARP2 is necessary to impact greatly on XRCC1 recruitment, PARP1 fulfils an additional role that is part of the same SSBR pathway but which is downstream of XRCC1/PNKP recruitment into chromatin and cannot be fulfilled by PARP2. That this role is within the XRCC1-dependent SSBR pathway is supported by the observation that PARP1 deletion does not further slow SSBR in *XRCC1^-/-^* RPE-1 cells (unpublished observations). In contrast to PARP1 and PARP2, we did not observe any impact of PARP3 on XRCC1 recruitment into oxidised chromatin. This may reflect that PARP3 primarily mono ADP-ribosylates proteins in response to SSBs, because the central BRCT1 domain of XRCC1 selectively binds poly (ADP-ribose) (7,43). The role fulfilled by of PARP3 activation at SSBs is currently unknown, but may involve the recruitment of one or more proteins that can bind mono-ADP ribosylated histone H2B (43).

In summary, we demonstrate here that surprisingly little ADP-ribosylation activity is required for the global recruitment of endogenous XRCC1 into oxidized chromatin, and we show that either PARP1 or PARP2 activity is sufficient for this process. It will now be of interest to determine whether these enzymes fulfil similar overlapping roles during the repair of other types of SSBs.

## Supplementary Material

Supplementary Data
