# Supplementary material for: Overlapping roles for PARP1 and PARP2 in the recruitment of endogenous XRCC1 and PNKP into oxidized chromatin
Source: Nucleic Acids Res. 2016 Dec 13;45(5):2546–57. doi: 10.1093/nar/gkw1246 (PMC5389470; doi:10.1093/nar/gkw1246)
Supplement: Supplementary Data [file gkw1246_Supp.pdf]

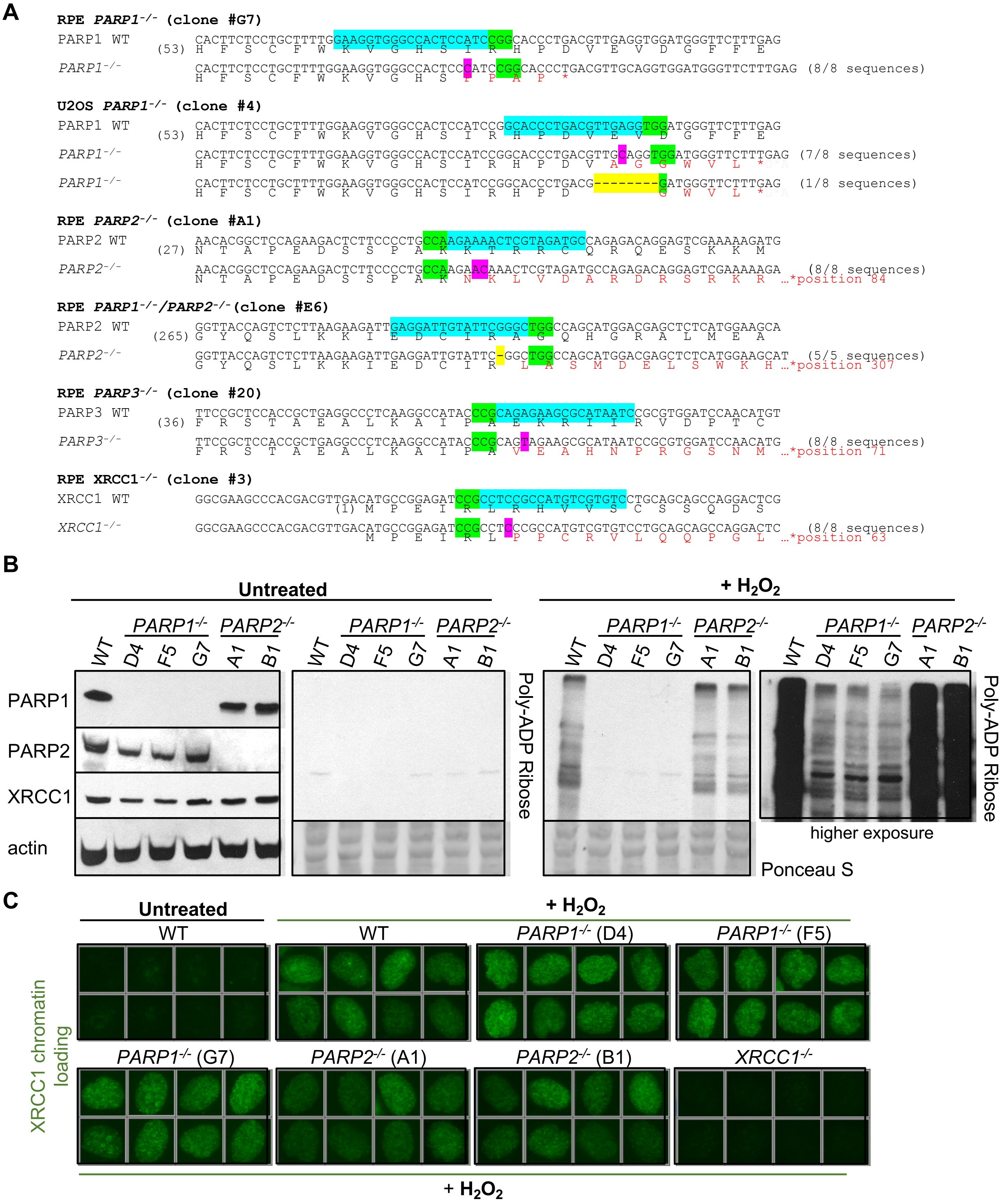


**Figure S1.** Characterization of gene-edited cell lines generated by CRISPR/Cas9. (**A**) Confirmation of gene editing was achieved by PCR amplification of genomic DNA spanning the gRNA target site in *PARP1*$^{-/-}$, *PARP2*$^{-/-}$, *PARP3*$^{-/-}$, *PARP1*$^{-/-}$/*PARP2*$^{-/-}$ and *XRCC1*$^{-/-}$ RPE-1 cells followed by subcloning and Sanger sequencing. Guides are indicated in WT sequences in blue and the associated PAM in green. Inserts and deletions in are in pink and yellow respectively. Frame-shifted amino acids are in red and the resulting stop codons indicated by an asterisk. The fraction of PCR sequences harbouring each indel are indicated. The *PARP1* mutations in *PARP1*$^{-/-}$ /*PARP2*$^{-/-}$ RPE-1 cells are the same as in *PARP1*$^{-/-}$ RPE-1 cells. (**B**) Wild type (WT), *PARP1*$^{-/-}$ (clones #D4, #F5, #G7) and *PARP2*$^{-/-}$ (clones #A1, #B1) cell lines untreated or treated with 10 mM $H_2O_2$ were immunoblotted for the indicated proteins or for poly-ADP-ribose by Western blotting. (**C**) The indicated cell lines were treated as above and then pre-extracted with detergent to remove non-chromatin bound proteins prior to fixation and immunostaining with XRCC1 antibody. Representative ScanR images are shown.

Supplementary Fig.2

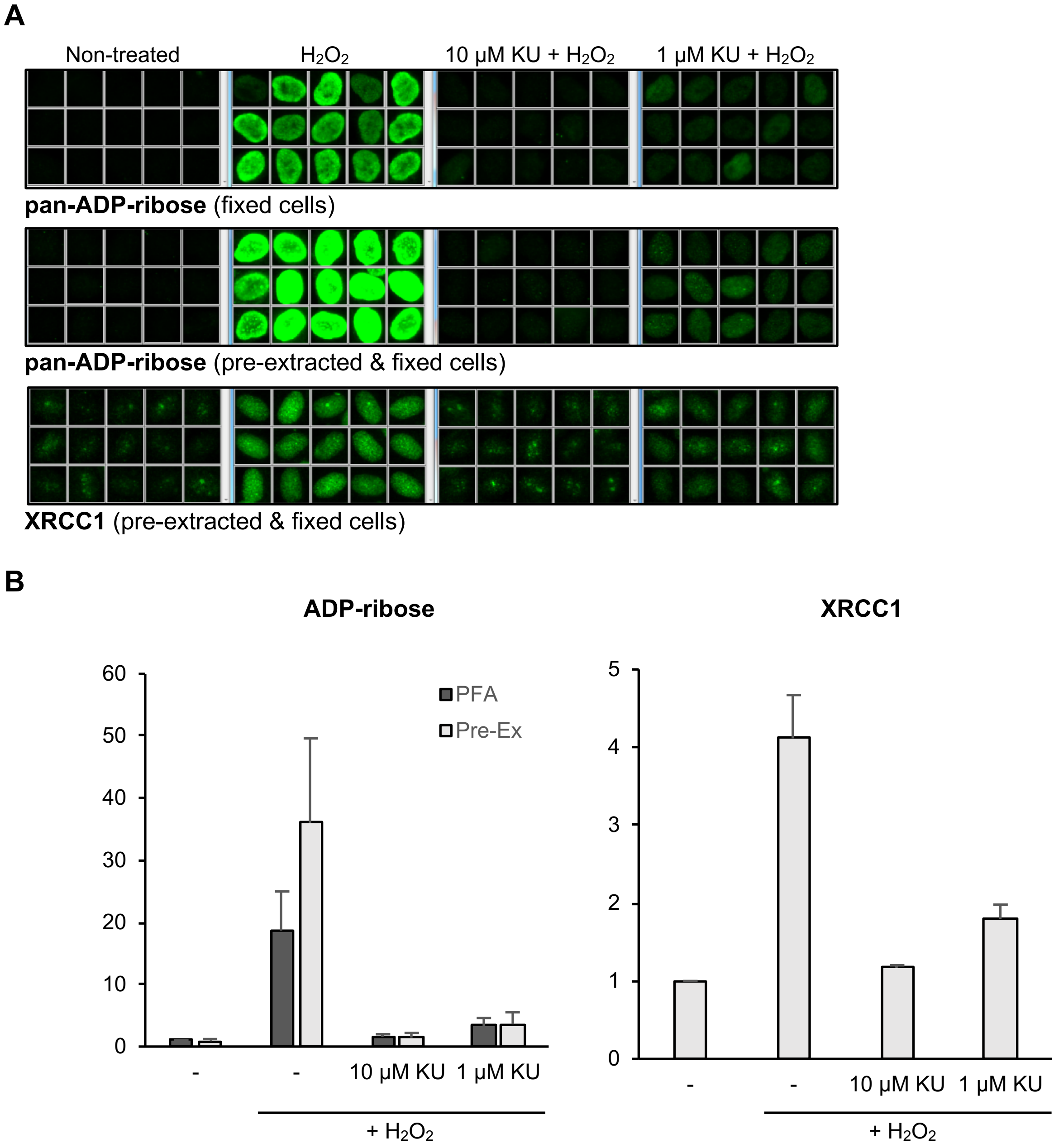


**Figure S2.** Impact of PARP inhibitor on ADP-ribosylation and loading of endogenous XRCC1 into oxidised chromatin. (**A**) Wild type (WT) RPE-1 cells were pre-incubated or not with 1 or 10 µM KU0058948 (KU) for 1 hour and subsequently treated for 10 min with 150 µM $H_2O_2$ on ice in the continued absence or presence of inhibitor, followed by rinsing PBS at room temperature. Cells were then pre-extracted or not (as indicated) with detergent to remove non-chromatin bound proteins and fixed prior to immunostaining with anti-pan-ADP-ribose binding reagent or anti-XRCC1 antibody. Representative ScanR images are shown. (**B**) Quantification of nuclear ADP-ribose (*left*) or XRCC1 (*right*) from >1000 cells per sample using Olympus ScanR acquisition and analysis software. Data are the mean (+/- SEM) of three independent experiments.

Supplementary Fig.3

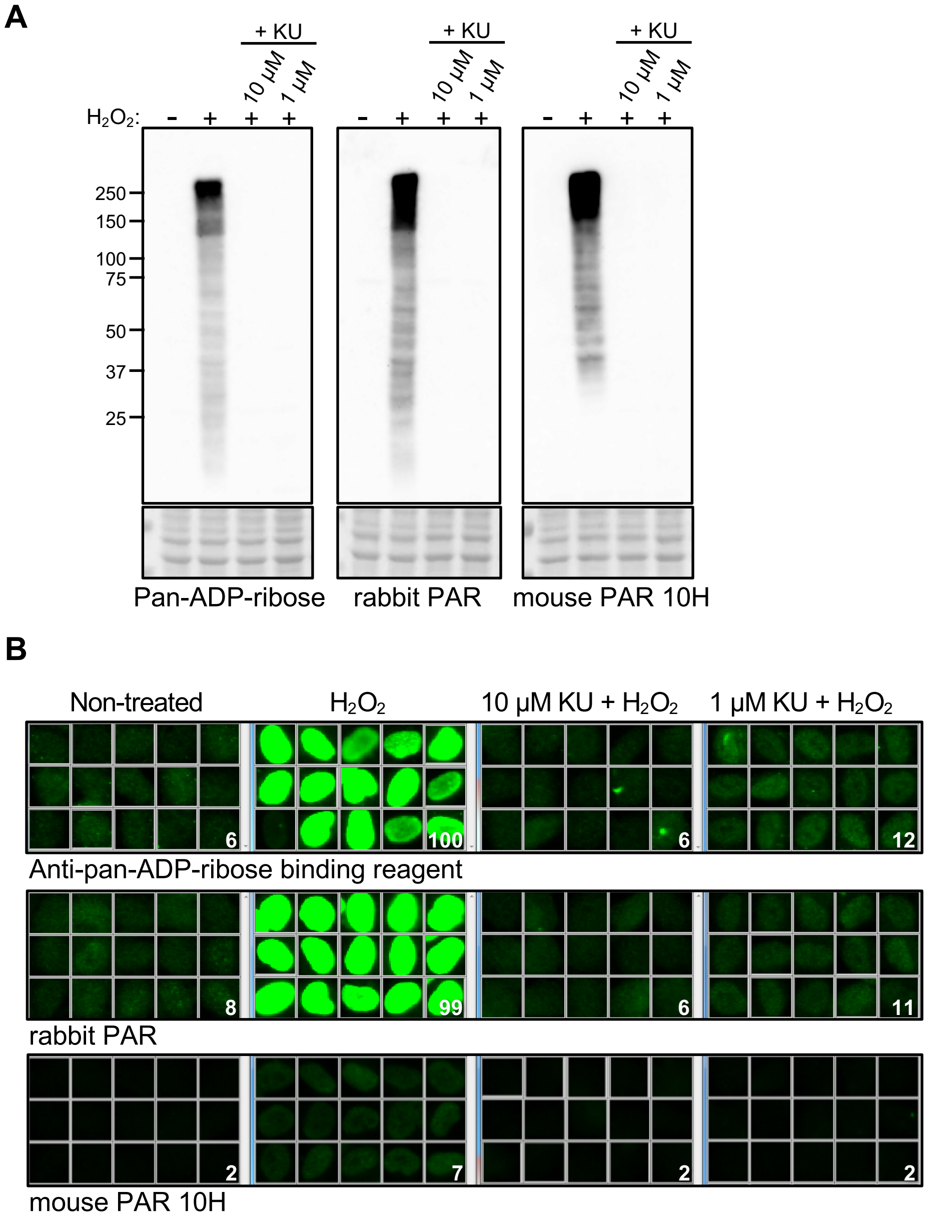


**Figure S3.** Impact of PARP inhibitor on ADP-ribosylation measured using different anti-(ADP-ribose) antibodies. Wild type RPE-1 cells were pre-incubated or not with 1 or 10 µM PARP inhibitor KU0058948 (KU) for 1 hour and subsequently treated for 10 min with 150 µM $H_2O_2$ on ice in the continued presence or absence of PARP inhibitors as indicated. ADP-ribosylated proteins were detected by Western blotting (**A**) or indirect immunofluorescence (**B**). Corner numbers are the mean nuclear ADP-ribose signal in >1500 cells relative (%) to the signal detected by anti-pan-ADP-ribose binding reagent in $H_2O_2$-treated cells (set at 100%). Data were quantified by Olympus ScanR acquisition and analysis software.

Supplementary Fig.4

A

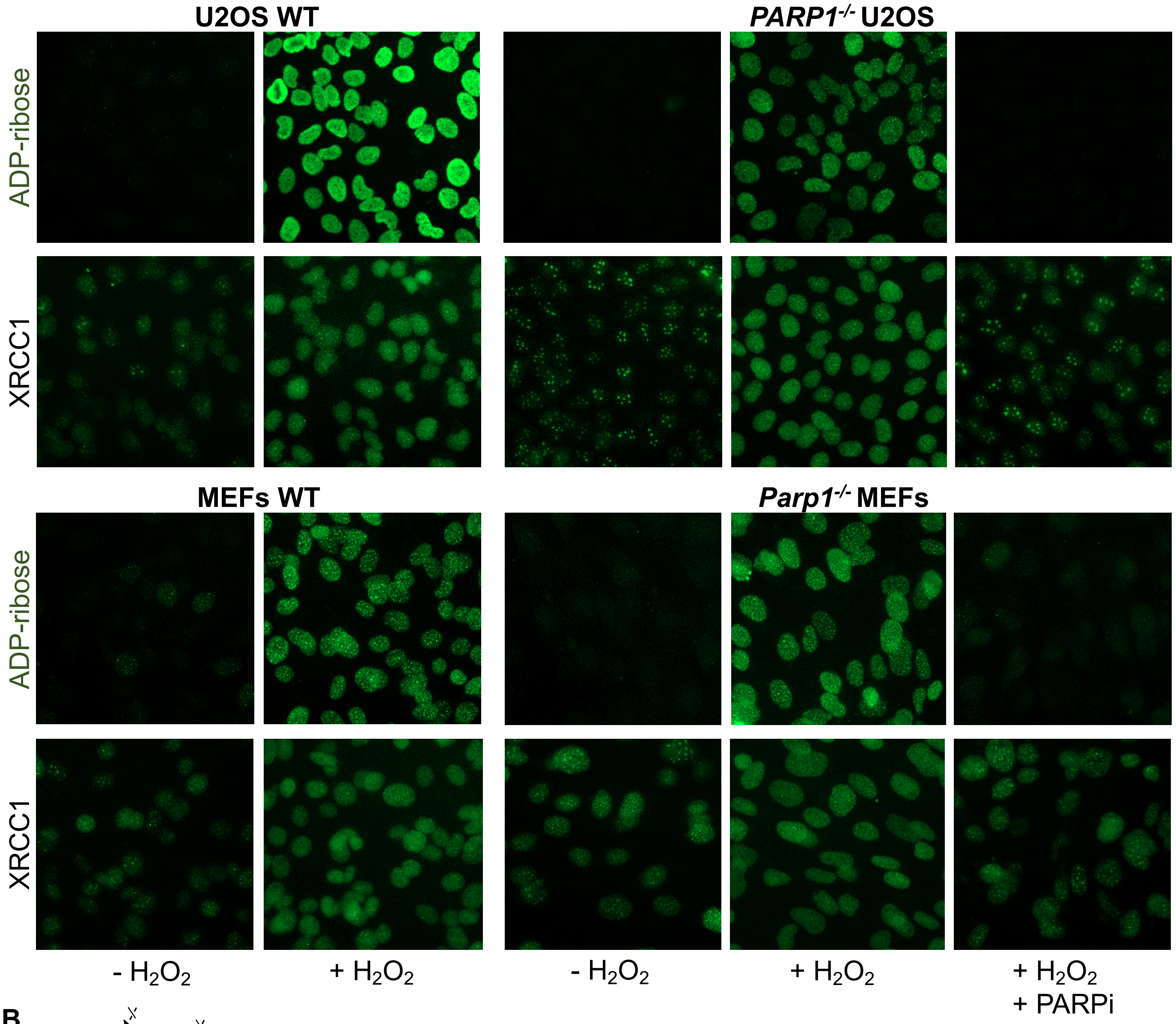


B

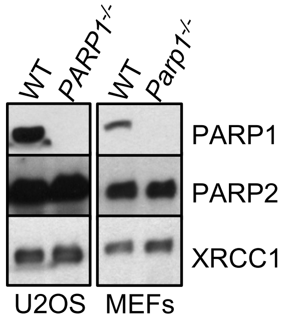


**Figure S4.** PARP-dependent recruitment of endogenous XRCC1 into oxidised chromatin in *PARP1*$^{-/-}$ MEFs and U2OS cells. (**A**) Wild type (WT) and *PARP1*$^{-/-}$ U2OS cells or mouse embryonic fibroblasts (MEFs) were treated or not with 10 mM $H_2O_2$ in combination with 10 µM Olaparib (PARPi) as indicated. Cells were pre-extracted with detergent prior to fixation and immunostaining with anti-pan-ADP-ribose binding reagent or anti-XRCC1 antibody. Representative Zeiss images are shown. (**B**) Indicated cell lines were analysed for the level of relevant protein by Western blotting.

Supplementary Fig.5

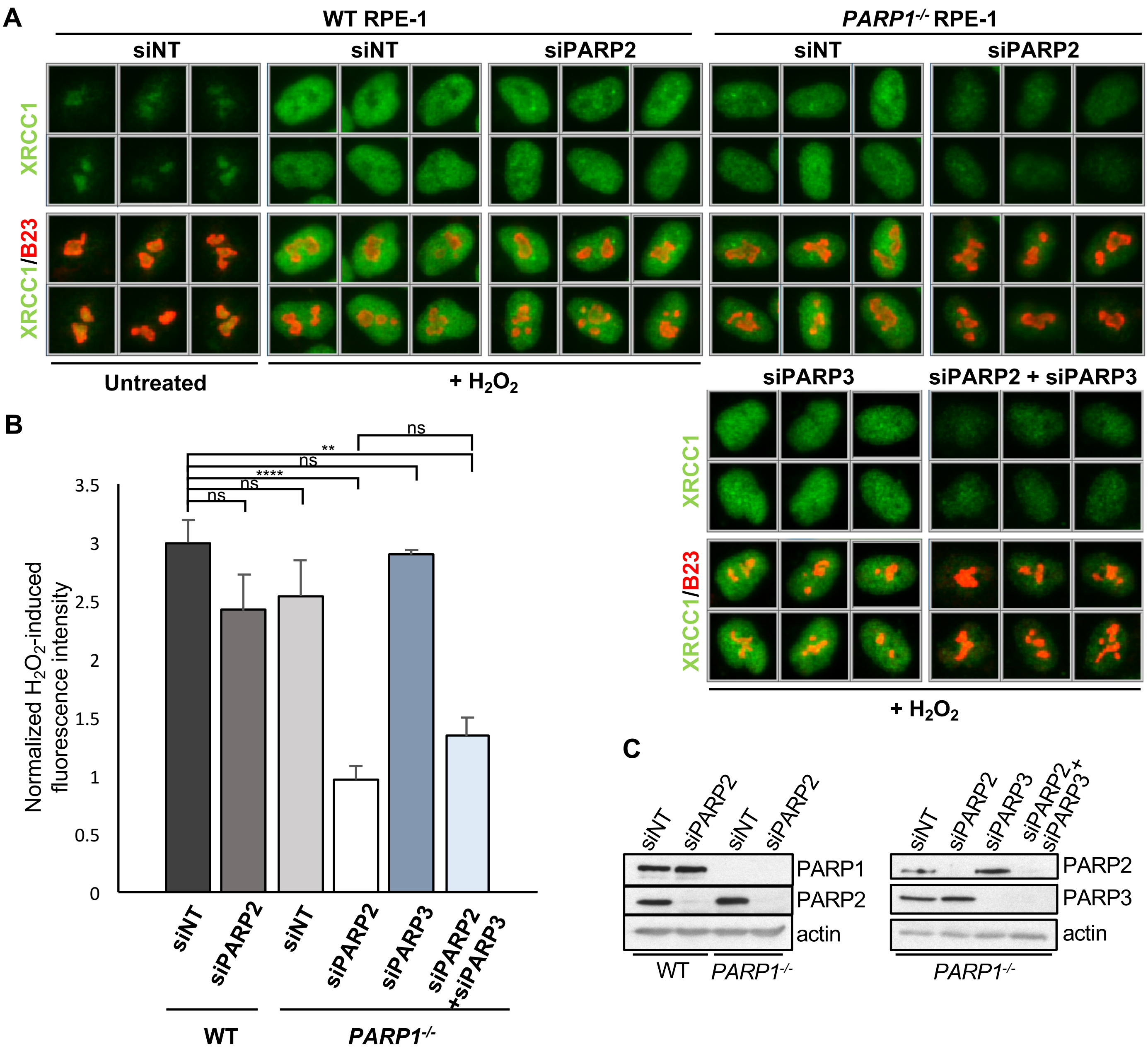


**Figure S5.** Overlapping roles for PARP1 and PARP2 in recruitment of XRCC1 into oxidised chromatin. (**A**) Wild type (WT) or *PARP1*$^{-/-}$ RPE-1 cells were transfected with non-targeting siRNA (siNT) or siRNA targeting PARP2 (siPARP2) and/or PARP3 (siPARP3) and 72 hr later treated or not with 400 μM $H_2O_2$ for 7 min. Cells were pre-extracted with detergent to remove non-chromatin bound proteins prior to fixation and immunostaining with anti-XRCC1 antibody and anti-B23 antibody. Representative ScanR images are shown. **(B)** Quantification of $H_2O_2$-induced chromatin bound XRCC1 (excluding nucleolar XRCC1 and corrected for XRCC1 background signal) in control and *PARP1*$^{-/-}$ cells mock-depleted (control siRNA) or depleted of PARP2 or PARP3 using appropriate siRNA and imaged by ScanR high content imaging in cells treated as in panel A. Data are represented as the fold over untreated control in each case. Data are the mean (+/- SEM) of three independent experiments with >1000 cells scored per sample in each experiment. Statistical significance was assessed by two tailed t-tests. Asterisks (**) indicate p-values of <0.01; ns - not significant. (**C**) Western blot demonstrating levels of PARP2 and PARP3 in the cells employed above.

Supplementary Fig.6

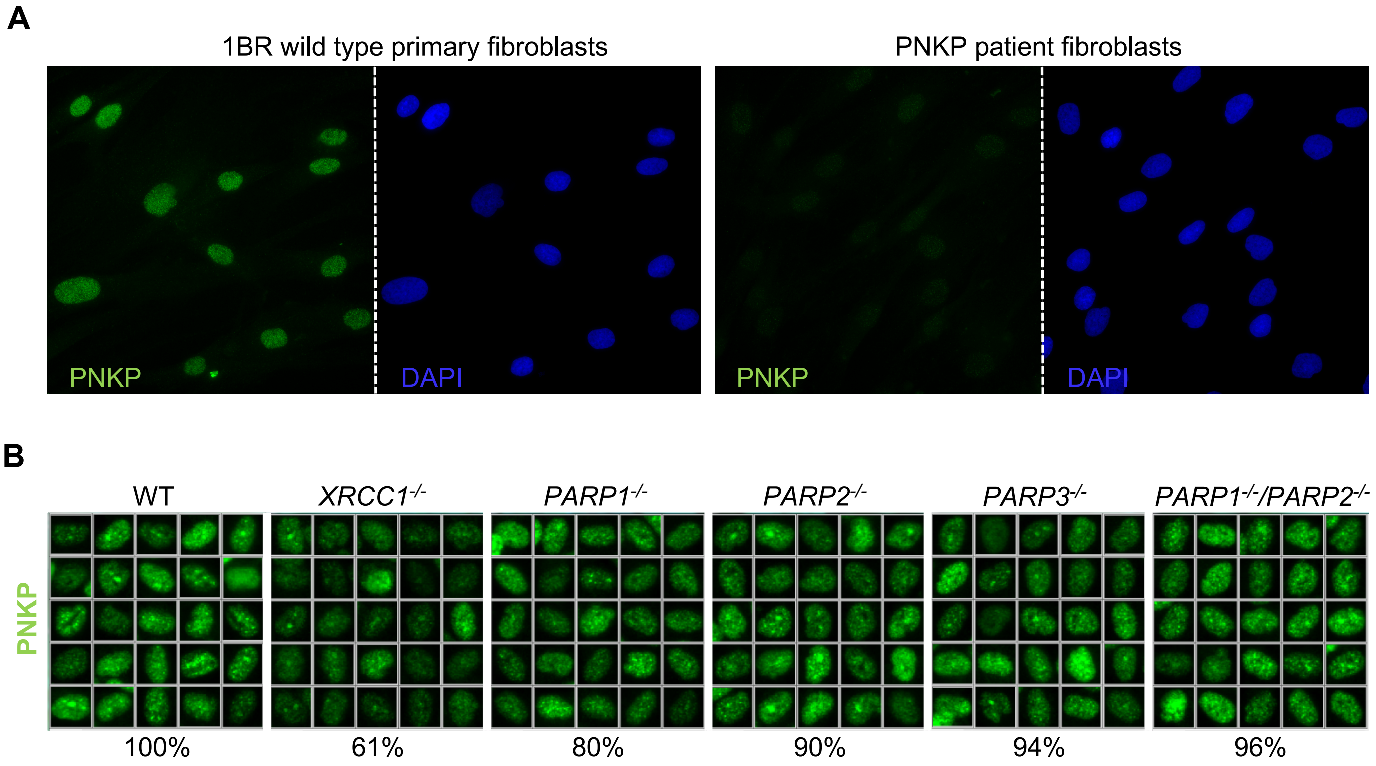


**Figure S6.** Comparison of total PNKP levels. (**A**) Representative Zeiss images showing wild type 1BR fibroblasts or PNKP patient fibroblast fixed and immunostained with anti-PNKP antibody (green) and DAPI (blue). (**B**) Representative ScanR images of indicated gene-edited RPE-1 cells showing total levels of PNKP (green). Numbers (%) are the mean PNKP signal in >2000 cells relative to the signal detected by anti-PNKP antibody in WT cells (set at 100%).

| Cell line | PCR primer (forward) | PCR primer (Reverse) | gRNA locus |
|---|---|---|---|
| *PARP1*$^{-/-}$ | CTTCTAGTCGCCCATGTTTGATGG | TACACACCTGTCACTCCTCCAGC | *PARP1*$^{-/-}$ |
| *PARP2*$^{-/-}$ | CTGGGCGAGAGAGTGAGACT | CCTGGCATACCATCTTGCTT | *PARP2*$^{-/-}$ |
| *PARP3*$^{-/-}$ | AGGGCCCTGAGAAGAAGAAG | GATGTCTGGGGTCAGGAGAA | *PARP3*$^{-/-}$ |
| *PARP1*$^{-/-}$ /*PARP2*$^{-/-}$ | GAAGCTGACAGTGGCACAAA | CTGCTGCTGATGGATACAGG | *PARP2*$^{-/-}$ |
| *XRCC1*$^{-/-}$ | GACCCCATACTCTACCTCATCC | CATTAATTCCCTCACGTCTTCC | *XRCC1*$^{-/-}$ |

**Supplementary Table 1.** Oligonucleotide sequences employed for PCR amplification and subcloning of genomic CRISPR guide target sites for confirmation of gene editing by Sanger sequencing.
